# Near-Infrared Light-Responsive Hydrogels for Highly Flexible Bionic Photosensors

**DOI:** 10.3390/s23094560

**Published:** 2023-05-08

**Authors:** Rui Huang, Zhenhua Fan, Bin Xue, Junpeng Ma, Qundong Shen

**Affiliations:** 1 Key Laboratory of High-Performance Polymer Materials and Technology of MOE, Department of Polymer Science and Engineering, School of Chemistry and Chemical Engineering, Nanjing University, Nanjing 210023, China; 2 Collaborative Innovation Center of Advanced Microstructures, National Laboratory of Solid-State Microstructure, Department of Physics, Nanjing University, Nanjing 210093, China; 3 State Key Laboratory of Analytical Chemistry for Life Science, Nanjing 210023, China

**Keywords:** biomimetics, ionic hydrogel, flexible photosensors, photoelectric effect, near-infrared light

## Abstract

Soft biological tissues perform various functions. Sensory nerves bring sensations of light, voice, touch, pain, or temperature variation to the central nervous system. Animal senses have inspired tremendous sensors for biomedical applications. Following the same principle as photosensitive nerves, we design flexible ionic hydrogels to achieve a biologic photosensor. The photosensor allows responding to near-infrared light, which is converted into a sensory electric signal that can communicate with nerve cells. Furthermore, with adjustable thermal and/or electrical signal outputs, it provides abundant tools for biological regulation. The tunable photosensitive performances, high flexibility, and low cost endow the photosensor with widespread applications ranging from neural prosthetics to human–machine interfacing systems.

## 1. Introduction

Wearable devices are of critical importance for human motion detection [1,2], sensory prostheses [3,4], and soft robotics [5]. Conventional functional nanomaterial-based electronic devices have disadvantages of limited mechanical flexibility, high cost, and weak biocompatibility [6]. Great effort has been paid to endowing wearable devices with as many new functionalities as possible [7]. Among them, flexibility and biocompatibility, in particular, are being given much attention, which can not only meet the requirements of conforming to complex curved and dynamic surfaces, but also realize a seamless and compatible interface with uneven and soft tissues/organs, and reduce the adverse reactions and potential threats to the human body caused by the long-term retention of foreign substances [8,9]. Therefore, high flexibility and biocompatibility are much needed to develop advanced wearable electronics, especially for biological-related applications, such as sensory prostheses and brain/machine interfaces [6].

Hydrogels are hydrophilic polymer networks swollen with large amounts of water. Due to their excellent biocompatible and mechanical properties, hydrogels are widely used for drug delivery [10,11], tissue engineering [12,13], wound healing [14,15], and bioelectronics [16,17]. In fact, hydrogel bioelectronics has become one of the leading fields of flexible electronics [18,19]. Classic wearable electronics achieve signal transduction using electrons as carriers, while our nerve sensory system is reliant on ionic species to transmit and process information. Thus, the hydrogels making use of ions as the signal carrier are more in line with the information transmission law of biological systems. Contributing to large amounts of water, which can dissolve ions, the hydrogels have great potential to mimic sensory systems to work and communicate with nerve cells in a natural way. Simulating the natural communication system of biological tissues is beneficial to closely interface and communicate with biological systems. Hydrogels, as ionic skin, have attracted attention and become a hotspot in recent years [20,21]. Vision is the most important way for us to collect abundant external information. However, even though hydrogels allow for high-level mechanical matching and biocompatibility with cells/tissues, as their direct integration may lead to suboptimal performance [18], their utilization for direct wireless photo-stimulation of neurons remains unexplored. To the best of our knowledge, a hydrogel as a bionic photosensor has not been reported yet.

Herein, we aim to demonstrate near-infrared light-responsive hydrogels for bionic photosensitive sensory. Mimicking the biological visual system, our sensors operate by light-driven ionic flux. This occurs by streaming anions, creating a net charge imbalance. Hydrogel photosensors allow for sensing near-infrared light, which is converted into a sensory signal that can communicate with nerve cells. In addition, it supports customized sensing signals by adjusting its composition to output tunable thermal and/or electrical signals.

## 2. Materials and Methods

### 2.1. Chemicals and Biochemicals

Hyaluronic acid (HA) with a molecular weight of 400 kDa was purchased from Tianjin Xiensaopude Technology Co., Ltd (Tianjin, China). Sodium ascorbate was purchased from Shanghai Meryer Chemical Technology Co., Ltd. (Shanghai, China). N-Hydroxysuccinimide (NHS) was purchased from Shanghai Yuanye Bio-Technology Co., Ltd. (Shanghai, China). 1-Ethyl-(3-dimethylaminopropyl) carbodiimide hydrochloride (EDC) was purchased from Shanghai Bide Pharmaceutical Technology Co., Ltd. (Shanghai, China). Manganese chloride anhydrous was purchased from Energy Chemical (Anqing, China). Zinc chloride, iron(III) chloride, and dopamine hydrochloride were purchased from Macklin (Shanghai, China). TRIS was purchased from Solarbio (Beijing, China). Phosphate-buffered saline (PBS) was purchased from Gibco (Grand Island, NY, USA). C-FOS antibody was purchased from Abcam (Cambridge, UK). Fluorescein isothiocyanate (FITC)-labeled secondary antibody and calcium ion fluorescence probe Fluo-4 AM were purchased from Biyuntian Biotechnology Co., Ltd. (Shanghai, China). Rat pheochromocytoma cell line (PC12) was purchased from KeyGen BioTECH (Nanjing, China). The indium tin oxide-coated polyethylene terephthalate (ITO-PET) film was purchased from South China Xiangcheng Technology Co., Ltd. (Yiyang, China). The polyimide film was purchased from Shenzhen Zhenpai Adhesive Co., Ltd. (Shenzhen, China).

### 2.2. Preparation of Hydrogel Sensors

Synthesis of HA-Dopa: HA-Dopa was synthesized on the basis of EDC/NHS-coupled amide bond reaction between the carboxylic acid group of HA and the amine group of dopamine [22]. First, 1 g of HA (MW: 400 kDa) was dissolved in deionized water. Then, 2.95 g of sodium ascorbate, 3.82 g of EDC, and 2.30 g of NHS were added to the HA solution and stirred for 1 h. The pH was adjusted to about 7.8 with 1 mol/L NaOH solution. The reaction mixture was sealed and blown with nitrogen for about 15 min to remove oxygen. Next, 2.97 g of dopamine was transferred into the EDC/HA/NHS solution to conjugate dopamine to HA. The reaction was conducted overnight at room temperature under magnetic stirring. The conjugate was purified by dialysis against excess Milli-Q water using a dialysis tubing with a molecular weight cutoff of 3.5 kDa. The final product was lyophilized and stored at 4 °C.

Formation of HA-Dopa-Mn^2+^ (Tris-HCl) hydrogel: First, 30 mg of HA-Dopa was dissolved in 45 µL of Tris-HCl buffer (pH = 8), and 5 µL of MnCl_2_ solution (2.4 mol/L) was added for cross-linking. According to a 2:1 molar ratio of dopa to metal ion, HA-Dopa-Zn^2+^ (Tris-HCl) hydrogel was prepared in the same way as HA-Dopa-Mn^2+^ (Tris-HCl) hydrogel. HA-Dopa-Fe^3+^ (Tris-HCl) hydrogel was prepared in accordance with a 3:1 molar ratio of dopa to metal ion.

Formation of HA-Dopa-Mn^2+^ (PBS) hydrogel: First, 30 mg of HA-Dopa was dissolved in 45 µL of PBS solution, and 5 µL of MnCl_2_ solution (2.4 mol/L) was added for crosslinking.

Formation of HA-Dopa (Tris-HCl) hydrogel: First, 30 mg of HA-Dopa was dissolved in 50 µL of Tris-HCl buffer (pH = 8).

Formation of HA-Dopa (Tris-HCl, with 500 μg/mL of PPy) hydrogel: First, 30 mg of HA-Dopa was dissolved in 50 µL of Tris-HCl buffer (pH = 8, with 500 μg/mL of polypyrrole particles). Polypyrrole was synthesized as reported in [23].

Formation of HA-Dopa-Mn^2+^ (Tris) hydrogel: First, 30 mg of HA-Dopa was dissolved in 45 µL of Tris solution, and 5 µL of MnCl_2_ solution (2.4 mol/L) was added for crosslinking. HA-Dopa-Zn^2+^ (Tris) hydrogel was formed in the same way.

Synthesis of BSA-HA-Dopa: BSA-HA-Dopa was synthesized on the basis of an EDC/NHS-coupled amide bond reaction between the carboxylic acid group of HA and the amine group of BSA. First, 400 mg of BSA and 400 mg of HA-Dopa were dissolved in 20 mL of deionized water. Then, 0.383 g of EDC and 0.230 g of NHS were added to the solution to crosslink the BSA and HA-Dopa. The hydrogel was transferred into the deionized water to achieve swelling equilibrium for 24 h, during which time the deionized water was refreshed > 6 times to remove the unreacted reactants.

Formation of BSA-HA-Dopa-Mn^2+^ (Tris) hydrogel: BSA-HA-Dopa was soaked in Tris buffer (1 mol/L) and then in MnCl_2_ solution (0.24 mol/L) to ensure that the final molar ratio of catechol and Mn^2+^ in the hydrogel was 1:2. Lastly, the hydrogel was washed with deionized water.

### 2.3. Fabrication of the Photo-Responsive Devices

**Figure 1:**
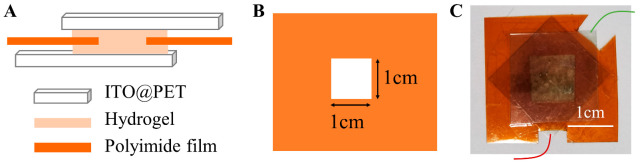
(**A**) Schematic diagram of hydrogel device. (**B**) A 1 cm^2^ hole was cut in the middle of polyimide film. (**C**) Image of hydrogel device.

The hydrogel was thinned and flattened, and the thickness was controlled at about 300 μm. The photo-responsive devices were prepared using indium tin oxide-coated polyethylene terephthalate (ITO-PET) films as the upper and lower electrodes of the hydrogel. A polyimide film was placed between the upper and lower electrodes as insulation material to prevent short-circuit. A 1 cm^2^ hole was cut in the middle to provide hydrogel contact with two electrodes (Figure 1).

### 2.4. Photoelectric and Photothermal Measurements

The photoelectric measurement was carried out using a CHI 660E electrochemical workstation (CH Instruments, Inc., Austin, TX, USA). The photothermal effect was tested using a FOTRIC thermal imager (Laserwave Optoelectronics Technology Co., Ltd., Beijing, China). For the fabricated device, its upper surface temperature was detected using an infrared imager, and its open-circuit voltage was simultaneously tested using an electrochemical workstation. Near-infrared light (808 nm, 1.26 W/cm^2^) was applied with an LWIRPD-1F type laser. The NIR light was fixed at a distance of 2–3 cm from the device.

### 2.5. Light Stimulation of the Nerve Cells

Calcium flow into cytoplasm: The photosensor was sterilized under an ultraviolet lamp overnight. PC12 cells were seeded in the cell culture dishes with quartz bottoms, and then cultured overnight until the cells adhered to the wall. Then, the photosensor was carefully attached to the top of the cell. Intracellular calcium-ion fluorescence imaging was performed using a Leica SP8 STED 3X confocal laser scanning microscope. The near-infrared light (808 nm, 1.26 W/cm^2^) was on for 90 s during the image time.

Immunofluorescence of c-FOS in the cells: The cells were seeded in cell culture dishes with quartz bottoms, and then cultured overnight until the cells adhered to the wall. The photosensor was carefully stuck on the top of the cell, and then irradiated with an infrared laser (808 nm, 1.26 W/cm^2^) for 120 s. After 1 h of illumination, the culture medium was replaced with 4% paraformaldehyde in PBS to fix the cell. The cells were fixed for 30 min, and then washed with PBS for 10 min each time for three times. The c-FOS antibody was diluted 1:1000 in the blocking solution (5% albumin dissolved in PBS), and then added to the cell culture dish for 4 °C overnight. The cells were washed with PBS three times (10 min/time). FITC secondary antibody (1:250 diluted in sealing solution) was added, and the cells were put in the dark for 1 h, before being washed again for the same washing condition. DAPI solution (1 μmol/L) was added, and then the cells were washed after 3 min. The samples were imaged using a confocal laser scanning microscope (Leica SP8 STED 3X).

## 3. Results

### 3.1. Design and Preparation of Biomimetic Photosensors

Light evokes neuronal signals through the retina to produce our perception of objects, movements, colors, etc. In the retina, the photoreceptor cells, i.e., rods and cones, are the first to respond to light [24]. When light is absorbed by the pigment in a photoreceptor, a G protein is activated, causing biochemical reactions. Consequently, nucleotide-gated cation channels close in the membrane [25]. Intuitively, light triggers the movement of ions across the cell membrane, resulting in a different charged state of the cells. To mimic the biological visual system, the photosensors operate by light-driven ion flux. The thermal gradient can drive the motion of ions to create a net charge imbalance. Such heat-to-electricity conversion is known as the ion Seebeck effect [26]. Herein, we utilize light to drive ionic flux in the hydrogels though the coupling of photothermal and thermoelectrical (Seebeck) effects. To achieve the free diffusion of anions, we constrain the motion of metal cations by binding them to the dopa groups on the polymer chain. Thus, the absorption of light initiates the separation of opposite charges within the hydrogel and sets up the transmembrane electric potential difference.

**Figure 2:**
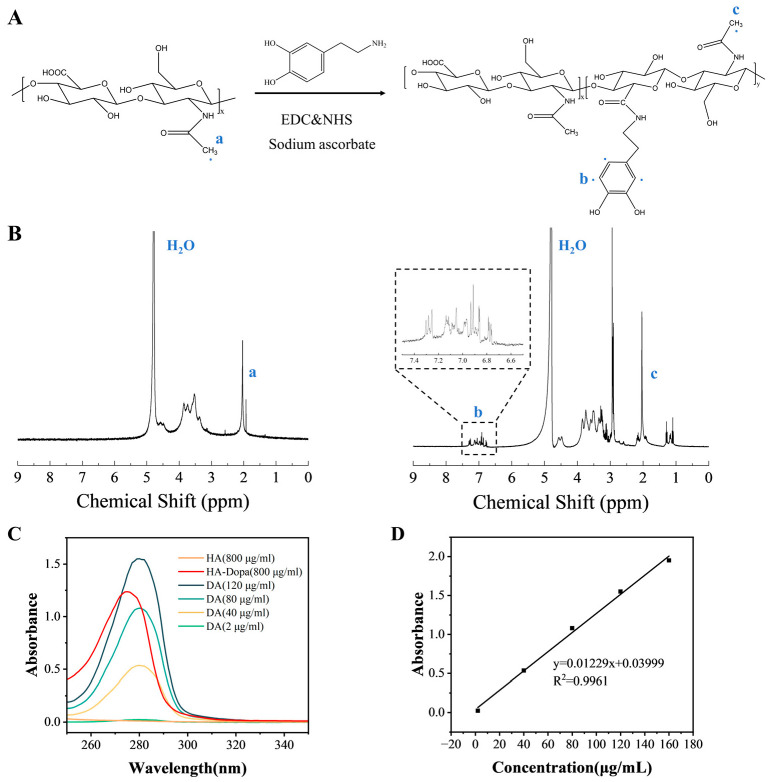
Structural characterization of dopamine-modified HA (HA-Dopa). (**A**) Synthetic route of HA-Dopa. (**B**) ^1^H-NMR spectrum of HA and HA-Dopa in D_2_O. Alkyl proton peaks (2 ppm, CH_3_^−^) were observed in the ^1^H-NMR spectrum of HA, which is indicated with “a”. Aromatic proton peaks (6.7–7.4 ppm, C_6_H_3_(OH)_2_^−^) of the dopa groups were observed in the ^1^H-NMR spectrum of HA-Dopa, which is indicated with “b”. Alkyl proton peaks (2 ppm, CH_3_^−^) were observed in the ^1^H-NMR spectrum of HA-Dopa, which is indicated with “c”. (**C**) UV absorbance spectra of HA, HA-Dopa, and dopamine (DA). (**D**) Linear regression line constructed with different mass concentrations of dopamine.

The polymer chains containing catechol groups were synthesized first. Dopamine-modified hyaluronic acid (HA-Dopa) was obtained by chemical conjugation of the amino groups of dopamine with the carboxyl groups of HA using EDC/NHS as coupling reagents, followed by dialysis to remove residual reactant (Figure 2A). Aromatic proton peaks (6.7–7.4 ppm, C_6_H_3_(OH)_2_^−^) of the dopa groups were observed in the ^1^H-NMR spectrum of HA-Dopa (Figure 2B). In the ultraviolet spectrum, an absorption peak was found at 275 nm in the solution of HA-Dopa, while the peak was absent from HA (Figure 2C). For determining the graft content of dopa groups in HA-Dopa, a regression line composed of standard samples, i.e., the solutions of different mass concentrations of dopamine, was constructed. The graft content of dopa groups in synthesized HA-Dopa conjugate was estimated to be 12.2% (Figure 2D). The dopa group can coordinate with various metal ions. By adding metal ions, the coordination of metal ions and dopa groups can serve as crosslinking for polymer chains.

### 3.2. Photo-Response of the Hydrogel and Mechanism

**Figure 3:**
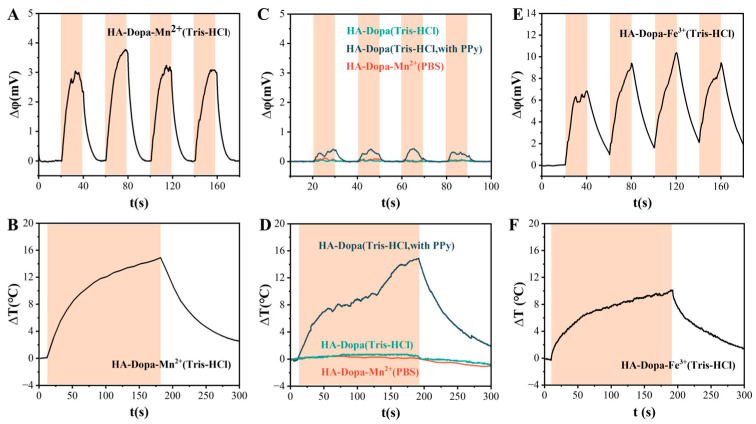
Photoelectric and photothermal performances of various hydrogel formulations. (**A**) Photoelectric and (**B**) photothermal performances of HA-Dopa-Mn^2+^ (Tris-HCl) hydrogel. (**C**) Photoelectric and (**D**) photothermal performances of HA-Dopa (Tris-HCl) hydrogel, HA-Dopa-Mn^2+^ (PBS) hydrogel and HA-Dopa (Tris-HCl, with 500 μg/mL of PPy) hydrogel. (**E**) Photoelectric and (**F**) photothermal performances of HA-Dopa-Fe^3+^ (Tris-HCl) hydrogel. The red-shaded area is with lighting on.

In order to simulate the sensing process of vision, the photoelectric conversion capability of the ionic hydrogels photosensor was evaluated. The dopa group can coordinate with various metal ions. The manganese ion-coordinated hydrogel (HA-Dopa-Mn^2+^ hydrogel) showed instant thermal and electrical responses to near-infrared light (Figure 3A,B). When manganese ions were not added to the hydrogel, the voltage change was extremely small and could be ignored (Figure 3C). For the hydrogel using PBS instead of Tris buffer, no obvious change in open-circuit voltage was observed under near-infrared light, even if manganese ion was added for crosslinking (Figure 3C). Neither of these hydrogels had an obvious photothermal effect (Figure 3D). For the photothermal hydrogel free from metal ions, even when polypyrrole particles with a significant photothermal effect were added to produce the same level of temperature rises on the light illumination (Figure 3D), its open-circuit voltage fluctuated with the light but was relatively small (Figure 3C). This small change was related to the presence of H^+^ and Cl^−^ in the hydrogel, and these two ions did not have coordination constraints in the hydrogel. It can be seen that the photothermal effect is a necessary condition for generating a photoelectric effect. The open-circuit voltage generated by the ion concentration gradient depends on the existence of thermal effect. If there is no thermal gradient to drive the ion movement, the voltage cannot be generated. Obviously, this process is inseparable from the ion Seebeck effect, i.e., the creation of a voltage across a material subject to a temperature gradient. The voltage originates from the diffusion of mobile charge carriers transported by the heat flux [27].

The ionic separation under the temperature gradient of hydrogel could be manipulated by the interaction between the networks and the metal ions, i.e., a strong metal-coordination interaction existed between the catechol in the HA-Dopa chains and the cations, which influenced the movement of the cations in the system. We tested the photovoltage effect of the iron ion-coordinated hydrogel. It can be seen that the photovoltage effect was stronger than that of the manganese ion-coordinated hydrogel (Figure 3E). The compositional dependence on the voltage change for these complex polymer hydrogels can be interpreted by considering the interactions between dopa and coordinated ions. This may be mainly because the crosslinking strength of iron ions and catechol was higher than that of manganese ions and catechol [28]. The iron ion-coordinated hydrogel also showed a significant photothermal effect (Figure 3F).

**Figure 4:**
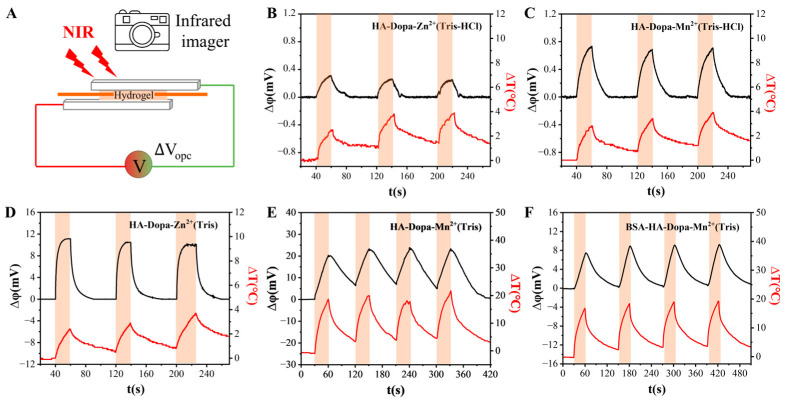
The change in open-circuit voltage and temperature of hydrogel. (**A**) Testing schematic diagram of photo-response performance of hydrogel photosensors. (**B**) HA-Dopa-Zn^2+^ (Tris-HCl) hydrogel. (**C**) HA-Dopa-Mn^2+^ (Tris-HCl) hydrogel. (**D**) HA-Dopa-Zn^2+^ (Tris) hydrogel. (**E**) HA-Dopa-Mn^2+^ (Tris) hydrogel. (**F**) BSA-HA-Dopa-Mn^2+^ (Tris) hydrogel. The red-shaded area is with lighting on.

To further illustrate the relationship between the thermal and electrical signals induced by light, we recorded changes in temperature and voltage simultaneously (Figure 4A). Both temperature and voltage rose when the light was applied and decreased when the light was removed. The manganese ion crosslinked hydrogel produced a higher photovoltage than zinc ion-coordinated hydrogel when other components were the same, consistent with the larger crosslinking strength of manganese ion and catechol than zinc ion and catechol [29] (Figure 4B–E). The photovoltage of zinc ion-coordinated hydrogel and manganese ion-coordinated hydrogel was improved significantly when there was no HCl in the hydrogel (Figure 4B–E). This may be because the hydrogen ions had an interference effect on the coordination between the metal ions and the dopa groups or because the free-moving hydrogen ions canceled out part of the anion concentration gradient generated by chloride ions.

**Figure 5:**
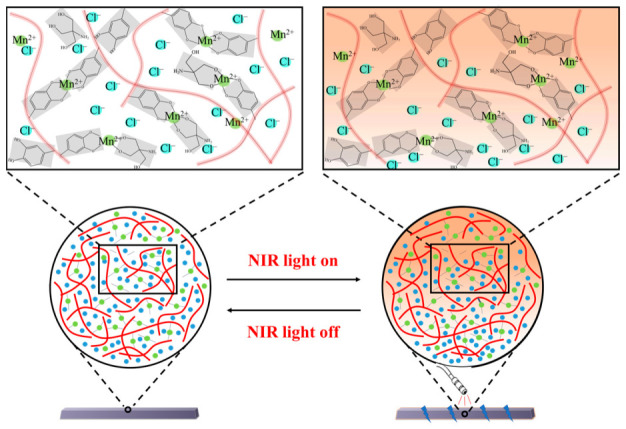
Schematic diagram of an ionic concentration gradient-driven voltage generator triggered by NIR light.

On the basis of the above data analysis, a schematic diagram of the photovoltaic mechanism is shown in Figure 5. Under light irradiation, a certain temperature gradient is present in the hydrogel, whose composition determines the temperature rise level. The plane of illumination of the hydrogel has a higher temperature than the other plane. Due to the constraint of coordination, the manganese ion has weak mobility in the system. Therefore, the diffusive mobile charge carriers driven by heat flux are mainly chloride ions. In the hydrogels, photovoltage is formed when chloride ions are distributed in concentration gradients from the plane of illumination to the other plane. Furthermore, as covalent bonds are stronger than coordination bonds, we modified the hydrogel with BSA to form covalent crosslinking and tested its photovoltaic generation ability. It exhibited an obvious near-infrared response (Figure 4F). The hydrogel photosensors demonstrated tunable crosslinking density and strength without losing optical response performance.

### 3.3. Signal Communication between the Photosensor and Nerve Cells

**Figure 6:**
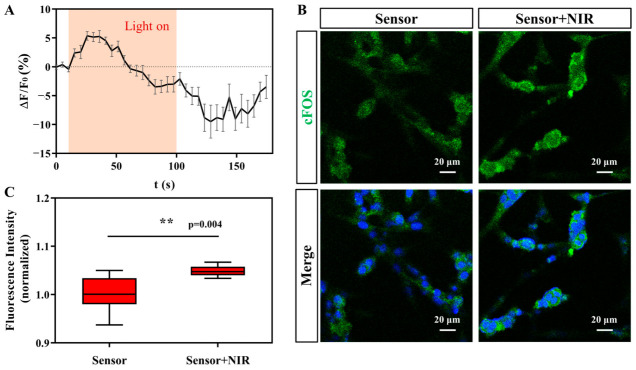
Light-induced stimulation of nerve cells by hydrogel photosensor. (**A**) Changes in fluorescence intensity of intracellular calcium ions after NIR light stimulation. (**B**) Expression of c-FOS in PC12 cells; the scale bar is 20 μm. (**C**) Relative c-FOS immunofluorescence intensity of the cells in different groups.

In order to further study the signal communication between the hydrogel photosensor and the nerve cells, confocal laser scanning microscopy was used to image the intracellular calcium ion fluorescence and fluorescent-labeled c-FOS protein of cells stimulated by the hydrogel photosensor under the irradiation of near-infrared light. Calcium ions are the second messenger of nerve cell communication. When heat, electricity, and other physical stimuli are exerted on the cells, the calcium ion channel on the membrane surface opens, and the calcium ions flow into the cytoplasm and regulate various physiological functions of the cells [29]. Therefore, the change in calcium concentration can indicate the response of the nerve cells to stimulation. BSA-HA-Dopa-Mn^2+^ (Tris) hydrogel was used as photosensor here. The photosensors were placed on top of the nerve cells to ensure full contact. When the light illuminated the system, the emission intensity of the calcium ion fluorescence probes increased rapidly (Figure 6A). This indicates that the calcium ions flowed into the cytoplasm, and the cells responded to the stimulation of the photosensors.

In addition, we evaluated whether the photosensors could activate the expression of c-FOS protein, which is the transcription product of the immediate early gene c-FOS, a marker of neuronal activation [30]. In our study, both groups of nerve cells were in contact with the photosensors. For the cells cultured with the photosensor, the fluorescence of c-FOS was upregulated after light irradiation (Figure 6B,C), consistent with the upregulation of calcium fluorescence after the cells received the photosensor signal.

## 4. Discussion

Conventional functional nanomaterial-based electronic devices are limited in terms of their mechanical flexibility and weak biocompatibility. Hydrogel bioelectronics have gained considerable attention owing to their high-level tissue-matching biomechanics and biocompatibility. Currently, a large number of hydrogel bioelectronics are focused on the development of bionic skin operated by pressure-driven ion flux [20,21]. Wireless sensors can provide the advantages of being wearable, real-time, and long-distance in vivo sensing [31]. For biomimetic photosensitive tissue, the application of hydrogel bioelectronics in wireless optoelectronic nerve prostheses remains unexplored. On the one hand, hydrogels, as conductive materials, have photosensitive properties but cannot output photoelectric stimulation. For example, Dong et al. developed a photosensitive stretchable conductive polymer hydrogel as an artificial nerve [16]. Under near-infrared light irradiation, the photothermal effect of the conductive hydrogel further promoted the movement of charged ions in the conductive hydrogel, enhanced the conductivity of the conductive hydrogel, and promoted the transmission of bioelectrical signals in mice with nerve injury. On the other hand, hydrogels are mostly used as flexible conductive substrates to assist traditional rigid photoelectric functional materials to realize phototransduction and transmission. So far, as an optoelectronic bio-interface integrated with poly(3,4-ethylenedioxythiophene)/poly(styrene sulfonate) (PEDOT:PSS), the hydrogel was advantageously used for the photo-stimulation of cells [32]. To achieve photoelectric conversion, ZnO/P3HT integrated with PEDOT:PSS hydrogel impaired its flexibility and restricted in vivo application. Photosensors fully composed of a hydrogel can maximize the advantages of flexibility and biocompatibility, while achieving animal-like ionic signal transmission. In this study, we demonstrated that highly flexible photosensors fully composed of hydrogels could achieve direct communication with nerve cells without any other accessory materials. The fully hydrogel-composed photosensors could facilitate the ever-close integration of artificial photosensitive systems with the human body, potentially blurring the boundary between humans and machines.

We showed that the hydrogel-based photosensors performed a photoelectric conversion function under the illumination of near-infrared light. By coupling the photothermal effect with the ionic Seebeck effect, our hydrogel photosensors enabled the light-driven ionic flux-to-electricity conversion. Thermoelectric materials based on the ionic Seebeck effect for heat harvesting have received substantial attention [26,33]. NaOH/PVA hydrogels with giant negative thermopower were obtained via synergistic coordination and hydration interactions [34]. The mechanism is based on coordination and hydration interactions under a temperature gradient to contribute to the thermoelectric effect of NaOH/PVA hydrogels. Similarly, with coordination as the basis for ion binding, the mechanism of our photosensor can be further studied in detail in the future. Compared to conventional ionic thermoelectric devices, our photosensors can directly convert light into ion currents, expanding the range of energy transduction. By streaming anions and creating a net charge imbalance, our photosensors also play a role as thermoelectric materials for heat harvesting, or as piezoelectric materials for force sensing beyond light perception. In other words, they have the potential to be developed into a multifunctional “biological tissue”.

More importantly, the hydrogels allow for high-level mechanical matching and biocompatibility with cells/tissues. As their direct integration may lead to suboptimal performance, their use is challenging for effective photo-stimulation of neurons [18]. Here, we put the photosensor directly in contact with the nerve cells, and the near-infrared light could communicate wirelessly with the cells. The hydrogel photosensors could transmit near-infrared light signals to the nerve cells, whose calcium influx increased rapidly, while the expression level of c-FOS protein was increased. This provides a powerful means for instant communication with nerve cells. The influx of Ca^2+^ brings pulse signals that contain biological information [35]. Similarly to the fundamentals of vision, the hydrogel photosensors may convert visible light into signals that can be recognized by optic neurons to form neural impulses that are transmitted to the visual cortex to form visual information. Therefore, our hydrogel has the potential to be used as a visual prosthesis to bring light perception to people who have lost their vision.

Highly flexible bionic photosensors can potentially be used against retinal degeneration diseases, such as age-related macular degeneration and retinitis pigmentosa. The operational wavelength of the photosensors in this study is within the near-IR range. For patients with age-related macular degeneration, the remaining healthy photoreceptors will not be activated, while the degenerated retinal parts will be stimulated after implanting the photosensor. The photosensors fully composed of the hydrogels are transparent, which is beneficial for retinal implants, whether via subretinal or epiretinal implantation. More interestingly, the near-infrared light-responsive hydrogel photosensors may supplement human night vision, or go so far as to serve as chips of human–machine interface systems. Toward a more viable solution for implants, there are several challenges in this study that need to be addressed. The current hydrogel photosensor needs to be nondegradable or ensure that the degradation takes place within the same time window as neural tissue regeneration. In addition, although the hydrogels are highly biocompatible materials, the number of ions in the hydrogels diffusing into adjacent cells/tissues needs to be controlled to minimize the interference with normal nerve function.

## 5. Conclusions and Outlook

We constructed photosensors operated by light-driven ion flux, mimicking a biological process. The ionic hydrogels with light-to-electricity conversion ability were demonstrated. The composition of the hydrogel photosensor affected its photoelectric conversion performance, and the mechanism of conversion was explained by the coupling between the photothermal and thermoelectric effects. The hydrogel photosensors exhibited impressive photoelectric and photothermal performance under near-infrared light. With respect to communication with nerve cells, the hydrogel photosensors could transmit near-infrared light signals to nerve cells, whose calcium ions increased rapidly, while the expression level of c-FOS protein increased. These results demonstrate the great potential of our hydrogels as biomimetic photosensors, providing new insight for the development of advanced flexible electronics for sensory prostheses and brain/machine interfaces. Using this proof of concept of flexible bionic photosensors, we look forward to its performance in an organism.

## Data Availability

Not applicable.

## References

[1] Ryu S., Lee P., Chou J.B., Xu R., Zhao R., Hart A.J., Kim S.-G. (2015). Extremely Elastic Wearable Carbon Nanotube Fiber Strain Sensor for Monitoring of Human Motion. ACS Nano.

[2] Yu X.-G., Li Y.-Q., Zhu W.-B., Huang P., Wang T.-T., Hu N., Fu S.-Y. (2017). A wearable strain sensor based on a carbonized nano-sponge/silicone composite for human motion detection. Nanoscale.

[3] Kim J., Lee M., Shim H.J., Ghaffari R., Cho H.R., Son D., Jung Y.H., Soh M., Choi C., Jung S. (2014). Stretchable silicon nanoribbon electronics for skin prosthesis. Nat. Commun..

[4] Liu H., Xu D., Hu B., Jiang J., Li M., Zhao D., Zhai W. (2021). Eco-friendly biogenic hydrogel for wearable skin-like iontronics. J. Mater. Chem. A.

[5] Zhu M., Biswas S., Dinulescu S.I., Kastor N., Hawkes E.W., Visell Y. (2022). Soft, Wearable Robotics and Haptics: Technologies, Trends, and Emerging Applications. Proc. IEEE.

[6] Wang L., Chen D., Jiang K., Shen G. (2017). New insights and perspectives into biological materials for flexible electronics. Chem. Soc. Rev..

[7] Wu H., Huang Y., Xu F., Duan Y., Yin Z. (2016). Energy Harvesters for Wearable and Stretchable Electronics: From Flexibility to Stretchability. Adv. Mater..

[8] Zhu B., Wang H., Leow W.R., Cai Y., Loh X.J., Han M.-Y., Chen X. (2016). Silk Fibroin for Flexible Electronic Devices. Adv. Mater..

[9] Won P., Kim K.K., Kim H., Park J.J., Ha I., Shin J., Jung J., Cho H., Kwon J., Lee H. (2021). Transparent Soft Actuators/Sensors and Camouflage Skins for Imperceptible Soft Robotics. Adv. Mater..

[10] Narayanaswamy R., Torchilin V.P. (2019). Hydrogels and Their Applications in Targeted Drug Delivery. Molecules.

[11] Sun Z., Song C., Wang C., Hu Y., Wu J. (2020). Hydrogel-Based Controlled Drug Delivery for Cancer Treatment: A Review. Mol. Pharm..

[12] Hasani-Sadrabadi M.M., Sarrion P., Pouraghaei S., Chau Y., Ansari S., Li S., Aghaloo T., Moshaverinia A. (2020). An engineered cell-laden adhesive hydrogel promotes craniofacial bone tissue regeneration in rats. Sci. Transl. Med..

[13] Spicer C.D. (2020). Hydrogel scaffolds for tissue engineering: The importance of polymer choice. Polym. Chem..

[14] Liang Y., He J., Guo B. (2021). Functional Hydrogels as Wound Dressing to Enhance Wound Healing. ACS Nano.

[15] Wang S., Zheng H., Zhou L., Cheng F., Liu Z., Zhang H., Wang L., Zhang Q. (2020). Nanoenzyme-Reinforced Injectable Hydrogel for Healing Diabetic Wounds Infected with Multidrug Resistant Bacteria. Nano Lett..

[16] Dong M., Shi B., Liu D., Liu J.-H., Zhao D., Yu Z.-H., Shen X.-Q., Gan J.-M., Shi B.-l., Qiu Y. (2020). Conductive Hydrogel for a Photothermal-Responsive Stretchable Artificial Nerve and Coalescing with a Damaged Peripheral Nerve. ACS Nano.

[17] Wang Z., Cong Y., Fu J. (2020). Stretchable and tough conductive hydrogels for flexible pressure and strain sensors. J. Mater. Chem. B.

[18] Yuk H., Lu B., Zhao X. (2019). Hydrogel bioelectronics. Chem. Soc. Rev..

[19] Li S., Cong Y., Fu J. (2021). Tissue adhesive hydrogel bioelectronics. J. Mater. Chem. B.

[20] Kim C.-C., Lee H.-H., Oh K.H., Sun J.-Y. (2016). Highly stretchable, transparent ionic touch panel. Science.

[21] Dobashi Y., Yao D., Petel Y., Nguyen T.N., Sarwar M.S., Thabet Y., Ng C.L.W., Scabeni Glitz E., Nguyen G.T.M., Plesse C. (2022). Piezoionic mechanoreceptors: Force-induced current generation in hydrogels. Science.

[22] Li Y., Qin M., Li Y., Cao Y., Wang W. (2014). Single Molecule Evidence for the Adaptive Binding of DOPA to Different Wet Surfaces. Langmuir.

[23] Barkade S.S., Pinjari D.V., Singh A.K., Gogate P.R., Naik J.B., Sonawane S.H., Ashokkumar M., Pandit A.B. (2013). Ultrasound Assisted Miniemulsion Polymerization for Preparation of Polypyrrole–Zinc Oxide (PPy/ZnO) Functional Latex for Liquefied Petroleum Gas Sensing. Ind. Eng. Chem. Res..

[24] Masland R.H. (2001). The fundamental plan of the retina. Nat. Neurosci..

[25] Berry M.H., Holt A., Levitz J., Broichhagen J., Gaub B.M., Visel M., Stanley C., Aghi K., Kim Y.J., Cao K. (2017). Restoration of patterned vision with an engineered photoactivatable G protein-coupled receptor. Nat. Commun..

[26] Li T., Zhang X., Lacey S.D., Mi R., Zhao X., Jiang F., Song J., Liu Z., Chen G., Dai J. (2019). Cellulose ionic conductors with high differential thermal voltage for low-grade heat harvesting. Nat. Mater..

[27] Zhao D., Martinelli A., Willfahrt A., Fischer T., Bernin D., Khan Z.U., Shahi M., Brill J., Jonsson M.P., Fabiano S. (2019). Polymer gels with tunable ionic Seebeck coefficient for ultra-sensitive printed thermopiles. Nat. Commun..

[28] Xu Z. (2013). Mechanics of metal-catecholate complexes: The roles of coordination state and metal types. Sci. Rep..

[29] Alawi K., Keeble J. (2010). The paradoxical role of the transient receptor potential vanilloid 1 receptor in inflammation. Pharmacol. Ther..

[30] Zhang Z., Ferretti V., Güntan İ., Moro A., Steinberg E.A., Ye Z., Zecharia A.Y., Yu X., Vyssotski A.L., Brickley S.G. (2015). Neuronal ensembles sufficient for recovery sleep and the sedative actions of α2 adrenergic agonists. Nat. Neurosci..

[31] Cui Y. (2017). Wireless Biological Electronic Sensors. Sensors.

[32] Han M., Yildiz E., Kaleli H.N., Karaz S., Eren G.O., Dogru-Yuksel I.B., Senses E., Şahin A., Nizamoglu S. (2022). Tissue-Like Optoelectronic Neural Interface Enabled by PEDOT:PSS Hydrogel for Cardiac and Neural Stimulation. Adv. Healthc. Mater..

[33] Akbar Z.A., Jeon J.-W., Jang S.-Y. (2020). Intrinsically self-healable, stretchable thermoelectric materials with a large ionic Seebeck effect. Energy Environ. Sci..

[34] Chen B., Chen Q., Xiao S., Feng J., Zhang X., Wang T. (2021). Giant negative thermopower of ionic hydrogel by synergistic coordination and hydration interactions. Sci. Adv..

[35] Malci A., Lin X., Sandoval R., Gundelfinger E.D., Naumann M., Seidenbecher C.I., Herrera-Molina R. (2022). Ca^2+^ signaling in postsynaptic neurons: Neuroplastin-65 regulates the interplay between plasma membrane Ca^2+^ ATPases and ionotropic glutamate receptors. Cell Calcium.

